# Simultaneous Quantification of 25-Hydroxyvitamin D_3_ and 24,25-Dihydroxyvitamin D_3_ in Rats Shows Strong Correlations between Serum and Brain Tissue Levels

**DOI:** 10.1155/2015/296531

**Published:** 2015-12-02

**Authors:** Ying Xue, Xin He, Huan-De Li, Yang Deng, Miao Yan, Hua-Lin Cai, Mi-Mi Tang, Rui-Li Dang, Pei Jiang

**Affiliations:** ^1^Institute of Clinical Pharmacy & Pharmacology, Second Xiangya Hospital, Central South University, Changsha 410011, China; ^2^School of Pharmaceutical Sciences, Central South University, Changsha 410011, China; ^3^Department of Pharmacy, Jining First People's Hospital, Jining Medical University, Jining 272000, China

## Abstract

While vitamin D_3_ is recognized as a neuroactive steroid affecting both brain development and function, efficient analytical method in determining vitamin D_3_ metabolites in the brain tissue is still lacking, and the relationship of vitamin D_3_ status between serum and brain remains elusive. Therefore, we developed a novel analysis method by using high performance liquid chromatography-tandem mass spectrometry (HPLC-MS/MS) to simultaneously quantify the concentrations of 25-hydroxyvitamin D_3_ (25(OH)D_3_) and 24,25-dihydroxyvitamin D_3_ (24,25(OH)_2_D_3_) in the serum and brain of rats fed with different dose of vitamin D_3_. We further investigated whether variations of serum vitamin D_3_ metabolites could affect vitamin D_3_ metabolite levels in the brain. Serum and brain tissue were analyzed by HPLC-MS/MS with electrospray ionization following derivatization with 4-phenyl-1,2,4-triazoline-3,5-dione (PTAD). The method is highly sensitive, specific, and accurate to quantify 25(OH)D_3_ and 24,25(OH)_2_D_3_ in animal brain tissue. Vitamin D_3_ metabolites in brain tissue were significantly lower in rats fed with a vitamin D deficiency diet than in rats fed with high vitamin D_3_ diet. There was also a strong correlation of vitamin D_3_ metabolites in serum and brain. These results indicate that vitamin D_3_ status in serum affects bioavailability of vitamin D_3_ metabolites in the brain.

## 1. Introduction

Vitamin D is implicated in a number of disorders such as cancer, immune function, and cardiovascular disease in addition to its established role in regulation of mineral balance and bone health [1–3]. Emerging research also suggests that vitamin D deficiency may play an important role in diseases of central nervous system (CNS), such as depression, Parkinson's disease, and epilepsy [4–6]. The ignorance of vitamin D status and profiles in brain tissue leads to a barrier in understanding the pathophysiological roles of vitamin D in CNS. Currently, the metabolism, storage, and functions of vitamin D in brain tissue remain ambiguous due to deficient information on the brain distribution of vitamin D metabolites and the correlation of vitamin D levels between serum and brain.

Vitamin D actually consists of two different compounds, vitamin D_3_ and vitamin D_2_. A nutritionally adequate amount of vitamin D_3_ is usually biosynthesized in the skin upon irradiation of 7-dehydrocholesterol by ultraviolet light, and it is also absorbed from the diet [7]. The serum levels of vitamin D_2_ (which is derived solely from plant sources) and its metabolites are usually less than one-tenth of those of vitamin D_3_ and its metabolites [8]. Thus, the quantification of vitamin D_3_ and its metabolites in serum is widely used as a mean of assessing vitamin D status. Once in circulation, vitamin D_3_ is converted to 25-hydroxyvitamin D_3_ (25(OH)D_3_) in the liver, which is subsequently converted into biologically active 1alpha,25-dihydroxyvitamin D_3_ (1*α*,25(OH)_2_D_3_) in the kidney [7]. The half-life of 1*α*,25(OH)_2_D_3_ (only 4–8 h) is shorter compared to the half-life of 25(OH)D_3_ (2-3 weeks), and 25(OH)D_3_ is the best indicator of vitamin D_3_ status because it reflects vitamin D supply by all sources well whereas the levels of 1*α*,25(OH)_2_D_3_ are tightly regulated by parameters of mineral metabolism [9]. 25(OH)D_3_ is thought to be deactivated via conversion into 24,25(OH)_2_D_3_ by 25-hydroxyvitamin D 24-hydroxylase [10]. Although only 25(OH)D_3_ provide clinically relevant information, the quantitation of 24,25(OH)_2_D_3_ can provide important information of vitamin D metabolism in a research environment.

Lots of advances have been made towards the analysis of vitamin D_3_ metabolites in serum in recent years. Gas chromatography- (GC-) mass spectrometry (MS) has been applied to vitamin D_3_ metabolites quantification [11]. However the high temperatures used in GC analyses often result in the formation of pyro and isopropyl isomers of the metabolites and there is a risk of degradation of metabolites, which can be avoided by liquid chromatography- (LC-) MS/MS based methods [12]. Considering that immunoassays also suffer from poor accuracy, poor repeatability, and interference, a national dialogue on the measurement of vitamin D status led by the NIH Office of Dietary Supplements has identified LC-MS/MS methodologies as the preferred approaches [13]. However, the analysis of vitamin D_3_ metabolites is still a challenge due to the poor ionization efficiency caused by lacking of ionizable polar groups. Derivatization techniques have been developed to enhance the ionization efficiency of vitamin D_3_ metabolites to improve the detection response [14]. The representative Cookson-type reagent, 4-phenyl-1,2,4-triazoline-3,5-dione (PTAD) which is commercially available, can quantitatively react with* s-cis-diene* of vitamin D_3_ metabolites and reduce interferences [7, 15].

Since vitamin D signaling plays an indispensable role in brain function and development [4, 16, 17], it is significant to find out whether the vitamin D_3_ metabolites levels in brain tissue would change with those in serum when rats were given diet lacking vitamin D_3_. Investigations that study vitamin D_3_ metabolites levels in brain tissue and serum of rats fed with different vitamin D_3_ diet may provide a new insight to better understand the relationship of vitamin D_3_ status between peripheral circulation and CNS. However, previous analysis of vitamin D_3_ metabolites in serum exhibited low sensitivities [15]. Simpler extraction methods and more sensitive detection methods are, therefore, required. In this work, we studied rats fed with different levels of vitamin D_3_ diet. HPLC-MS/MS method was developed for simultaneous analyses of these compounds both in rat serum and brain samples. Data were analyzed to access the correlation of vitamin D_3_ metabolites levels in brain tissue and serum for the first time. We further investigated whether levels in brain tissue would synchronously change with the levels in serum of rats fed with different vitamin D_3_ diet.

## 2. Material and Methods

### 2.1. Chemicals

The water was purified using the 12 VDC RO+DI reagent grade water purification systems from AQUA solutions, Inc. (Jasper, Georgia, USA). HPLC-grade acetonitrile (AcN) and methanol (MeOH) were purchased from Merck KGaA (Darmstadt, Germany) and HPLC-grade formic acid (FA) was from ROE scientific Inc. (St. Newark, DE, USA). The standards of 25(OH)D_3_ and 24,25(OH)_2_D_3_ were purchased from ApexBio Technology LLC (Boston, MA, USA). Deuterated internal standard (IS) d6-25(OH)D_3_ was obtained from Sigma-Aldrich (St. Louis, MO, USA). PTAD obtained from Tokyo Chemical Industry Co. (Tokyo, Japan) was used as derivatization reagent.

### 2.2. Samples Collection

Six-week-old Sprague-Dawley rats were obtained from the Experimental Animal Center of the Second Xiangya Hospital. The rats were kept in a 12/12 light/dark cycle at ambient temperature (20−22°C) and 3 animals per cage. Rats were randomly assigned into 3 groups (6 in each): low vitamin D_3_ (LVD), normal vitamin D_3_ (NVD), and high vitamin D_3_ (HVD). All animals were fed with diets containing 10 000 IU/kg (HVD) or 1000 IU/kg (NVD) or 0 IU/kg (LVD) vitamin D_3_ (1 IU = 40 *μ*g) for 6 weeks. Blood was obtained by venipuncture and then centrifuged at 3000 ×g for 10 min to separate the serum and blood cells. After sacrifice, brains were removed from the skull on ice. Serum and brains were frozen on dry ice and stored at −80°C until analysis.

### 2.3. Sample Preparation

#### 2.3.1. Brain Samples

After being finely thawed, 1 mL of AcN and 10 *μ*L IS solution (containing d6-25(OH)D_3_ 100 ng/mL in AcN) were added to 90 mg of rat brain tissue, and the mixtures were homogenized using a tissue homogenizer avoiding light. After vortex for 5 min, the mixture was centrifuged at 4°C for 10 min at 15 000 ×g. The supernatant (800 *μ*L) was then transferred into another Eppendorf tube and subsequently dried under nitrogen gas. The remaining homogenate from different brain tissues was pooled as QC samples for method validation of brain. For derivatization, 100 *μ*L of PTAD solution (1 mg/mL in AcN) was added to the residue followed by 30 s of vortex and 3 min of centrifugation at 15 000 g under 4°C. The mixture was put in the room temperature for overnight reaction avoiding light throughout.

#### 2.3.2. Serum Samples

Sample preparation for rat serum was adapted from published methods for human serum [18]. Briefly, 200 *μ*L serum was added with 600 *μ*L AcN and 10 *μ*L IS solution (containing d6-25(OH)D_3_ 100 ng/mL in AcN). The mixtures were vortex-mixed for 3 min and centrifuged at 4°C for 10 min at 15 000 ×g. A batch of serum was mixed as QC samples for method validation of serum. The supernatant (650 *μ*L) was then transferred into another Eppendorf tube and subsequently dried under nitrogen. 100 *μ*L of PTAD solution (1 mg/mL in AcN) was added to the residue for derivatization. Then the mixtures were mixed, centrifuged, and reacted under room temperature overnight as described above.

### 2.4. Chromatography and Mass Spectrometry

Separation was performed using a Shimadzu LC-20AD chromatograph (Shimadzu Corporation, Kyoto, Japan). Samples were kept in the autosampler in vials at 4°C, and 5 *μ*L samples were injected on the column. The Thermo Accucore C18 column (2.6 *μ*m, 100 × 4.6 mm, Thermo Fisher Scientific Inc. Waltham, MA, USA) was kept at 35°C. Aqueous phase A was deionized water containing 0.1% FA as a modifier. Organic phase B was 100% MeOH. Starting gradient conditions were 39% A/61% B from 0 to 1 min, reaching 14% A/86% B at 2 min and maintaining for 5.5 min, then returned to 39% A/61% B at 8 min, and retained 3.5 min for equilibration. The flow rate was set at 0.3 mL/min. For MS/MS analysis, a QTRAP 4000 mass spectrometer was operated in electrospray ionization- (ESI-) positive ion multiple reaction monitoring (MRM) mode with the curtain gas set to 25 psi, ion spear voltage set to 5000 V, source temperature set to 600°C, ion source gas set to 170 psi, ion source gas set to 270 psi, declustering potential set to 80 V, entrance potential set to 10 V, and collision cell exit potential set to 10 V. Other compound specific settings were listed in Table 1.

### 2.5. Method Validation

#### 2.5.1. Preparation of Standard Curves and Linearity Range

By dissolving the analytes in AcN, stock solutions of 25(OH)D_3_ (1.0 mg/mL) and 24,25(OH)_2_D_3_ (0.2 mg/mL) were prepared, which were then further diluted in AcN to the appropriate concentrations for the preparation of calibration curve. The standard curve was prepared in 100% AcN that were analyzed within the same analytical run. The levels of brain tissue standard curve for 25(OH)D_3_ were 0.39, 0.98, 3.91, 5.86, 78.13, 195.31, 937.50, and 1250.00 ng/mL and for 24,25(OH)_2_D_3_ were 0.47, 0.78, 3.13, 7.81, 62.50, 93.75, 500.00, and 1000.00 ng/mL. The levels of serum standard curve for 25(OH)D_3_ were 0.10, 0.21, 1.04, 5.21, 52.08, 197.92, 833.33, and 1000.00 ng/mL and for 24,25(OH)_2_D_3_ were 0.25, 0.50, 2.08, 7.92, 50.00, 100.00, 200.00, and 333.33 ng/mL. To determine the linear range of the method, eight levels (*n* = 3, at each concentration level) of calibration samples were prepared and analyzed as mentioned above.

#### 2.5.2. Limit of Detection and Quantification

The limit of detection (LOD) and limit of quantification (LOQ) were defined as the peaks that give signal to noise ratios of 3 : 1 and 10 : 1, respectively, in triplicate.

#### 2.5.3. Precision and Accuracy

To determine the precision and accuracy, endogenous levels of vitamin D_3_ metabolites in brain and serum QC samples were analyzed. QC samples were prepared following the same procedure giving low, medium, and high concentrations of analytes. 800 *μ*L supernatants of brain homogenates or 200 *μ*L serum was spiked with 10 *μ*L of the IS solution and 10 *μ*L of specific standard solution to generate calibration levels covering a range of analytes (Table 3), respectively. The intraday precision and accuracy were calculated by analyzing QC samples at three concentrations (*n* = 5, at each concentration level) on the same day. The interday precision and accuracy were determined by analyzing the three concentrations in five replicates on three successive days. The precision was calculated as the coefficient of variance (CV) of the intraday and interday analytical results. As reported by previous studies, the accuracy was determined as recovery of each analyte in QC samples at three levels [15, 19]. Accuracy of each analyte was assessed by comparing the difference between QC samples and average levels of mixed blank samples with the added standard.

#### 2.5.4. Matrix Effect

For evaluation of matrix effect (ME), experiments were conducted according to previous work [20, 21]. The spiked samples were prepared by spiking known amounts of standards to 200 *μ*L extracted pooled serum or 800 *μ*L supernatants of brain homogenates. The added concentrations of analytes were the same as the QC samples. And calibrator solutions in AcN with the same levels of standards as the QC samples were also prepared (*n* = 5). The increase in the peak area ratios of the compounds was compared with the respective area ratio measured in calibrator solutions to which the same levels of standards had been added. The matrix effect was calculated as follows:

(1) $$\begin{array}{c} ME\left(\;\%\;\right)=100\times \left[\;1-\frac{\text{peak area ratio in spiked sample}-\text{peak area ratio in pooled sample}}{\text{peak area ratio in calibrator solution}}\;\right]. \end{array}$$

### 2.6. Statistical Analysis

Data acquisition was operated by Analyst 1.6.1 software (AB Sciex). The statistical analysis of the method validation results including calculation of mean, standard deviation, and coefficient of variance was performed using Microsoft Excel. Linear regression analysis using the least-squares method was used to evaluate the calibration curve of each analyte. Associations between analytes in tissue and serum were assessed using Pearson correlation coefficient. The ratio of brain and blood for analytes was assessed by one-way ANOVA and Dunnett's post hoc test with SPSS software (version 18.0). The cut-off for statistical significance was set at *P* < 0.05.

## 3. Results

### 3.1. Chromatography and Mass Spectrometry Conditions

**Figure 1:**
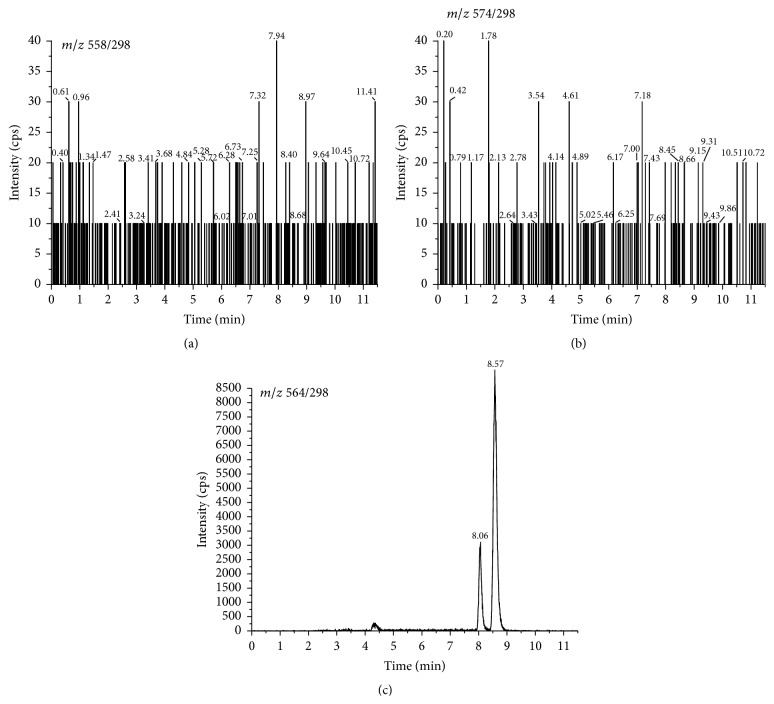
The representative MRM chromatograms for blank sample added with IS.

**Figure 2:**
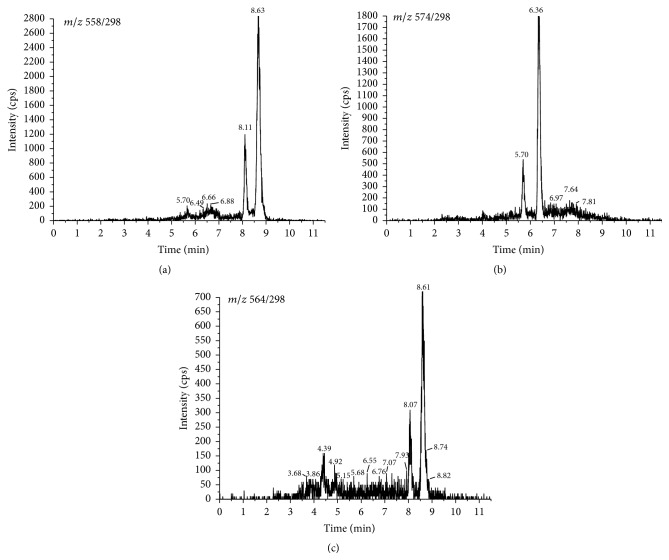
The representative MRM chromatograms for 25(OH)D_3_-PTAD (a), 24,25(OH)_2_D_3_-PTAD (b), and d6-25(OH)D_3_-PTAD (c) of serum.

**Figure 3:**
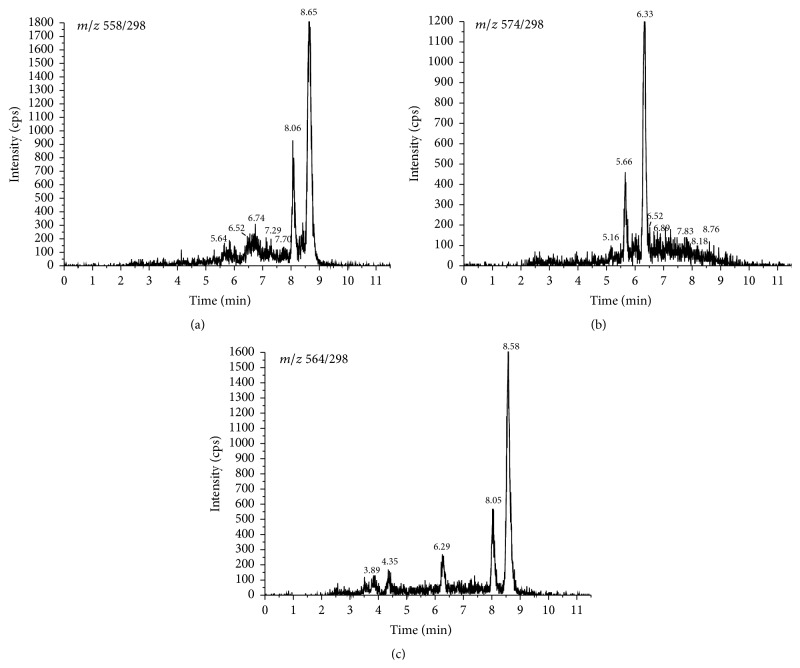
The representative MRM chromatograms for 25(OH)D_3_-PTAD (a), 24,25(OH)_2_D_3_-PTAD (b), and d6-25(OH)D_3_-PTAD (c) of brain tissue.

**Figure 4:**
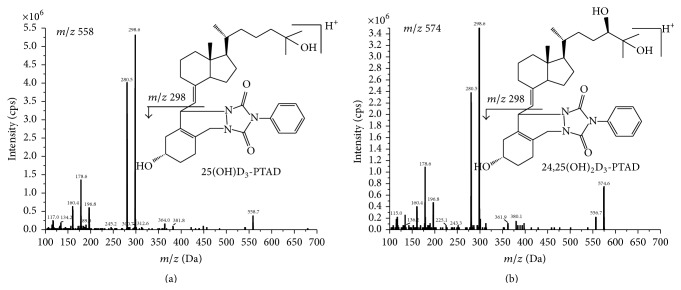
Production mass spectra of 25(OH)D_3_-PTAD (a) and 24,25(OH)_2_D_3_-PTAD (b).

According to previous reports, targeted analytes herein produced a much stronger signal in positive mode using the electrospray ion source. Thus, all the vitamin D metabolites were detected under MRM mode. The collision energy was optimized for each mass transition (Table 1). The dwell time established for each transition was 100 ms. The specificity of the method was determined by AcN added with IS. No interferences were observed at the retention time of 25(OH)D_3_-PTAD and 24,25(OH)_2_D_3_-PTAD (Figure 1). The representative MRM chromatograms for 25(OH)D_3_-PTAD, 24,25(OH)_2_D_3_-PTAD, and d6-25(OH)D_3_-PTAD in serum and brain tissue homogenates are shown in Figures 2 and 3. Production mass spectra for 25(OH)D_3_-PTAD and 24,25(OH)_2_D_3_-PTAD are shown in Figure 4.

### 3.2. Linearity, LOQ, Precision, Accuracy, and Matrix Effect

All standard curves showed good linearity both in brain homogenates and in serum. The equations of the standard curves, corresponding linear regression coefficients, and linear ranges were illustrated in Table 2. Since the targeted analytes are endogenous metabolites, the LOQs were determined by standard mixtures. The results of LOQs for each analyte were also illustrated in Table 2.

The data of intraday precision, interday precision, and recovery assays were summarized in Table 3 for all analytes both in brain and in serum. The precision was expressed by CVs ranged from 2.5% to 12.9% for intraday precision and from 1.2% to 3.3% for interday precision. Accuracy was determined by recovery. The values of standard addition accuracies and CVs in the QC samples were also shown in Table 3.

The matrix effect for 25(OH)D_3_ ranged from −3.1 to −5.3 in serum and ranged from −4.1 to −8.1 in brain. For 24,25(OH)_2_D_3_, the matrix effect ranged from −3.2 to −4.3 in serum and ranged from −3.6 to −6.6 in brain. The results suggested insignificant matrix effects in the present method.

### 3.3. Analysis of Vitamin D_3_ Metabolites in Both Serum and Brain Tissue

The HPLC-MS/MS method was used for the simultaneous determination of 25(OH)D_3_ and 24,25(OH)_2_D_3_ in rat brain tissue homogenates and serum. The results are summarized in Table 4. Serum levels of 25(OH)D_3_ and 24,25(OH)_2_D_3_ significantly increased from the LVD to the NVD group and were highest in the HVD group. In brain tissue, there was also a similar increase of vitamin D_3_ metabolites over the groups. Compared to the group of normal vitamin D supplement, there was no statistical difference of the 25(OH)D_3_ brain/serum ratio in different groups, as well as the 24,25(OH)_2_D_3_ brain/serum ratio. The brain and serum ratios of 25(OH)D_3_/24,25(OH)_2_D_3_ of different groups were also analyzed compared to the NVD group, and no statistical difference was found.

### 3.4. Associations of Analytes in Serum and Brain Tissue

**Figure 5:**
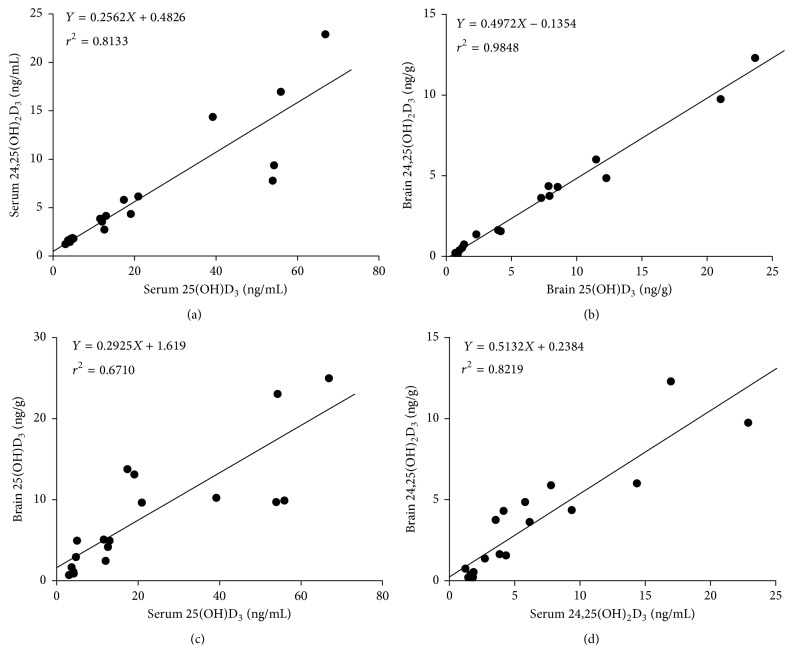
Correlation between serum 25(OH)D_3_ and serum 24,25(OH)_2_D_3_ (a), brain 25(OH)D_3_ and brain 24,25(OH)_2_D_3_ (b), serum 25(OH)D_3_ and brain 25(OH)D_3_ (c), and serum 24,25(OH)_2_D_3_ and brain 24,25(OH)_2_D_3_ (d).

Linear regression was used to analyze the levels of vitamin D_3_ metabolites between serum and brain tissue. Significant correlations were discovered between 25(OH)D_3_ and 24,25(OH)_2_D_3_ levels in serum (Figure 5(a), *r* ^2^ = 0.8133). Meanwhile, there were also linear correlations of 25(OH)D_3_ and 24,25(OH)_2_D_3_ levels in brain (Figure 5(b), *r* ^2^ = 0.9848). Similarly, linear regression analysis also indicated that 25(OH)D_3_ in serum and brain were highly correlated in the total samples (Figure 5(c), *r* ^2^ = 0.6710). The levels of 24,25(OH)_2_D_3_ in serum and brain were also significantly correlated (Figure 5(d), *r* ^2^ = 0.8219).

## 4. Discussion

In this study, we designed a new method for synchronous measuring of 25(OH)D_3_ and 24,25(OH)_2_D_3_ in brain tissue and serum. By using this method, we analyzed vitamin D_3_ metabolites in rats fed with different vitamin D_3_ doses. As far as we know, this is the first study showing that vitamin D_3_ metabolites in brain could be significantly affected by vitamin D_3_ diet and were strongly correlated with serum levels.

The developed LC-MS/MS method was proved to be highly sensitive, specific, and accurate to quantify the 25(OH)D_3_ and 24,25(OH)_2_D_3_ in animal brain tissue for the first time. The procedure described by Lipkie et al. involved a liquid/liquid extraction step to purify the analytes in rat soft tissues, while no satisfying recovery for 25(OH)D_3_ was achieved [22]. In our work, we employed a simple sample preparation procedure to attain the highly efficient extraction of vitamin D metabolites. Meanwhile, the use of PTAD, a Cookson-type reagent which can react with conjugated diene system of vitamin D metabolites, resulted in an approximately 100-fold increase in the analytical response. Aronov et al. researched the derivatization rates of 25(OH)D_3_ with PTAD at room temperature [15]. According to a pseudo-first-order kinetics model, *t*_1/2_ for 25(OH)D_3_ is shorter than 1 min. Thus >99% yield of derivatization products was achieved after overnight reaction at room temperature in our method. Furthermore, they also found that an increase in PTAD concentration to over 2 mg/mL led to decreased yield. Thus, 1 mg/mL PTAD was chosen in our work. Recent studies showed that two epimers,* 6S* and* 6R*, were produced by derivatization with PTAD because the reagent reacted with the* s-cis-diene* moiety from both the *α*- and the *β*-sides, and the ratio of 6*S*/6*R* was approximately 4 : 1 [23]. Accordingly, there were two peaks for each compound in the MRM ion chromatograms. In this case, the major peak for the* 6S*-isomer was used for integration and quantification.

As mentioned above, growing evidence has implicated that deficiency of vitamin D plays an important role in diseases of CNS [24, 25]. Thus, it is essential to find out the relationship between serum vitamin D_3_ status and its brain concentration. In this research, we evaluated the correlation of 25(OH)D_3_ and 24,25(OH)_2_D_3_ levels between serum and brain tissue of rats with different vitamin D_3_ intake for the first time. The results suggested that 25(OH)D_3_ status in brain tissue was highly correlated with 25(OH)D_3_ in serum of different groups. After giving rats vitamin D_3_ deficiency intake for 6 weeks, 25(OH)D_3_ serum levels decreased in parallel to a similar decrease of 25(OH)D_3_ in brain tissue, while, in the supplement group, 25(OH)D_3_ in brain tissue increased with its level in serum. Specific transport mechanisms have been proposed for the transportation of the circulating vitamin D metabolites to the CNS. In peripheral circulation, the majority of 25(OH)D_3_ tightly binds to vitamin D binding protein (DBP), forming vitamin D-DBP-complex [26, 27]. The transportation of vitamin D-DBP-complex is dependent on the molecules Megalin and Cubulin in microvessel endothelial cells of rat cerebral [28]. We speculated that Megalin-dependent transport in the choroid plexus could be important for the correlation between 25(OH)D_3_ in brain tissue and serum, and the expression of Megalin may change synchronously with the 25(OH)D_3_ status in serum to meet the demands of transportation. Furthermore, we found that the 24,25(OH)_2_D_3_ concentrations were highly correlated with 25(OH)D_3_ in serum in different groups, as well as in brain tissue. The results indicated that the catabolism of 25(OH)D_3_ into 24,25(OH)_2_D_3_ rose with increasing 25(OH)D_3_ concentrations, which were consistent with the findings of other investigators [29–31]. It also suggests that 24,25(OH)_2_D_3_, the most abundant 25(OH)D_3_ metabolite, in serum or brain tissue cloud serve as an alternative marker of vitamin D status and measurement of 24,25(OH)_2_D_3_ may provide clinically useful information pertaining to vitamin D status and supplementation. Meanwhile the determination coefficient for serum and brain 24,25(OH)_2_D_3_ was 0.8219. The strong correlations demonstrated in our research may provide a potential way to estimate the levels of 25(OH)D_3_ and 24,25(OH)_2_D_3_ in brain tissue from the data of serum concentrations.

## 5. Conclusion

The current study presented a novel HPLC-MS/MS method for simultaneous quantification of 25(OH)D_3_ and 24,25(OH)_2_D_3_ in serum and brain tissue of rats and proposed strong correlations of 25(OH)D_3_ and 24,25(OH)_2_D_3_ between brain tissue and serum in rats receiving different level of vitamin D_3_ for the first time. The results indicated that the levels of vitamin D metabolites in serum were closely related to those in brain, which may influence the employment of neural cells to vitamin D. Considering that vitamin D_3_ plays an important role in brain development, insufficient intake of vitamin D_3_ may negatively affect the function of CNS. Further research should continue to explore the alteration of vitamin D_3_ metabolites levels with different durations for vitamin D_3_ supplementation that may aid the clinicians in adjusting the length of time for vitamin D_3_ supplement to achieve optimum individual benefit.

## Figures and Tables

**Table 1:** Mass spectrometry conditions.

Analytes	MRM transition	CE	Dwell
	(*m*/*z*)	(V)	(ms)
25(OH)D_3_-PTAD	558/298	25	100
24,25(OH)_2_D_3_-PTAD	574/298	30	100
d6-25(OH)D_3_-PTAD	564/298	25	100

**Table 2:** Calibration statistics.

Analytes	Regression equation	*r* ^2^	Linear range	LOQ
			(ng/mL)	(ng/mL)
Serum				
25(OH)D_3_	*y* = 0.3841*x* − 0.0477	0.9981	0.10–1000.00	0.10
24,25(OH)_2_D_3_	*y* = 0.5848*x* − 0.4085	0.9992	0.25–333.30	0.25
Brain				
25(OH)D_3_	*y* = 0.9673*x*	0.9947	0.39–1250.00	0.10
24,25(OH)_2_D_3_	*y* = 0.861*x*	0.9979	0.47–1000.00	0.25

**Table 3:** Intraday and interday precision and recovery of rat brain homogenates and serum.

Analytes	ADD	Intraday		Interday	
	(ng/mL)	Accuracy %	CV %	Accuracy %	CV %
25(OH)D_3_					
Serum	7.5	98.1	3.5	97.4	1.5
	60.0	103.3	5.0	102.5	3.3
	120.0	93.7	4.5	92.8	1.2
Brain	0.5	99.3	12.9	102.5	2.7
	7.5	102.0	9.2	101.1	1.4
	11.3	99.3	2.5	101.8	1.3
24,25(OH)_2_D_3_					
Serum	7.5	98.2	4.3	97.5	3.3
	10.5	99.1	5.8	100.2	1.3
	21.0	98.0	7.1	98.0	1.9
Brain	1.1	100.9	6.5	103.9	1.7
	1.5	96.3	6.8	98.9	1.4
	3.0	96.3	4.8	98.7	1.5

**Table 4:** The levels of targeted analytes and ratio of 25(OH)D_3_/24,25(OH)_2_D_3_ in brain homogenates and serum.

Analytes	Brain	Serum	Brain/serum
	(ng/g)	(ng/mL)	
25(OH)D_3_			
LVD	1.01 ± 0.25^*∗∗*^	4.14 ± 0.72^*∗∗*^	0.26 ± 0.11
NVD	4.10 ± 1.16	14.61 ± 4.40	0.28 ± 0.07
HVD	13.34 ± 7.26^*∗∗*^	53.52 ± 8.89^*∗∗*^	0.25 ± 0.13
24,25(OH)_2_D_3_			
LVD	0.46 ± 0.24^*∗∗*^	1.13 ± 0.50^*∗∗*^	0.44 ± 0.17
NVD	2.73 ± 1.68	4.37 ± 0.96	0.60 ± 0.28
HVD	6.84 ± 2.84^*∗∗*^	13.52 ± 5.74^*∗∗*^	0.52 ± 0.08
25(OH)D_3_/24,25(OH)_2_D_3_			
LVD	2.64 ± 1.15	4.67 ± 2.97	—
NVD	1.80 ± 0.74	3.37 ± 0.61	—
HVD	1.99 ± 0.88	4.51 ± 1.77	—

Values are expressed as mean ± SD; *n* = 6 for each group. LVD: low vitamin D_3_ group; NVD: normal vitamin D_3_ group; HVD: high vitamin D_3_ group. ^*∗∗*^ *P* < 0.01 compared to the NVD group.
